# MicroRNA-141 and MicroRNA-200c Are Overexpressed in Granulosa Cells of Polycystic Ovary Syndrome Patients

**DOI:** 10.3389/fmed.2018.00299

**Published:** 2018-10-29

**Authors:** Tingting He, Yuan Liu, Yueyue Jia, Haiyan Wang, Xiao Yang, Gang Lu, Hongbin Liu, Yuhua Shi

**Affiliations:** ^1^Center for Reproductive Medicine, National Research Center for Assisted Reproductive Technology and Reproductive Genetics, Shandong University, Jinan, China; ^2^The Key Laboratory of Reproductive Endocrinology (Shandong University), Ministry of Education, Shandong Provincial Clinical Medicine Research Center for Reproductive Health, Jinan, China; ^3^Department of Obstetrics and Gynecology, Qilu Hospital of Shandong University, Jinan, China; ^4^Liaocheng People's Hospital, Liaocheng, China; ^5^Shandong Medical Imaging Research Institute Affiliated to Shandong University, Jinan, China; ^6^CUHK-SDU Joint Laboratory on Reproductive Genetics, School of Biomedical Sciences, The Chinese University of Hong Kong, Hong Kong, China

**Keywords:** polycystic ovary syndrome, miR-200c, miR-141, granulosa cells, pregnancy complications

## Abstract

Polycystic ovary syndrome (PCOS) is one of the most common endocrine disorders in reproductive-aged women, affecting 6–8% of women and characterized by hyperandrogenism, ovulatory dysfunction, and polycystic ovarian morphology. Accumulating evidence demonstrates that different microRNAs (miRNAs) expressions may contribute to the pathogenesis of PCOS. Therefore, the goal of this study is to compare the expression levels of miR-141 and miR-200c in granulosa cells isolated from PCOS patients and also evaluate their predictive values for pregnancy complications. First, RNA extraction, reverse transcription, and reverse transcription-polymerase chain reaction (RT-PCR) were performed to assess the expression levels of miR-141 and miR-200c in granulosa cells isolated from 62 PCOS patients and 61 controls. Second, according to each mean of miR-141 and miR-200c measured values in all patients, PCOS, and controls were divided into low-expression group and high-expression group to better evaluate their predictive values for pregnancy complications. Significantly elevated expressions of miR-141 and miR-200c were observed in PCOS patients compared with the controls (*p* < 0.001 and *p* = 0.002, respectively). Furthermore, PCOS patients had a significantly increased incidence of pregnancy complications in low-expression groups of miR-141 and miR-200c (*p* = 0.007 and *p* = 0.002, respectively). Our findings demonstrated that the expressions of both miR-141 and miR-200c were significantly increased in PCOS patients, which might contribute to the pathogenesis of PCOS. PCOS patients had an increased risk of pregnancy complications in low-expression groups of both miR-141 and miR-200c.

## Introduction

Polycystic ovary syndrome (PCOS) is one of the most common endocrine disorders in reproductive-aged women, affecting 6–8% of women and characterized by hyperandrogenism, ovulatory dysfunction, polycystic ovarian morphology(1, 2). It is also associated with metabolic disorders, including insulin resistance, obesity, and diabetes (3). It has been estimated that environmental and genetic factors may play a vital role in the pathogenesis of PCOS (4, 5).

MicroRNAs (miRNAs) are short (20–24 nucleotides) non-coding RNAs that regulate post-transcriptional gene expression and play an important role in biological processes, including glucose and lipid metabolism (6–9). Altered expressions of miRNAs have a close link with wide ranges of human disease, such as cancer, metabolic, and viral pathogenesis (10). Burgeoning number of evidence has highlighted that different miRNAs expression profiles exist in granulosa cells derived from PCOS patients and controls, which may contribute to the pathogenesis of PCOS (11–13). In PCOS rat ovaries, miR-141 is associated with promoting the development of PCOS by inhibiting cell growth and facilitating cell apoptosis (14, 15). MiR-200c has recently been related to pregnancies complicated by pre-eclampsia and preterm labor, indicating that it may cover a crucial role in pregnancy (16). However, no study has investigated whether the expressions of miR-141 and miR-200c are altered in the granulosa cells of PCOS patients.

Therefore, this study was to research underlying molecular mechanisms of PCOS and their clinical outcomes.

## Materials and methods

### Patients enrollment

Ovarian granulosa cells were collected from 62 PCOS patients and 61 controls who underwent *in vitro* fertilization (IVF) or intracytoplasmic sperm injection (ICSI) at the Center for Reproductive Medicine, Shandong University between Oct 2015 and June 2016. This study was approved by the ethics committees of Reproductive Medicine Center of Shandong University. Formal written consent was obtained from each patient. A long protocol was used for ovarian stimulation in all patients. Diagnosis of PCOS was carried out according to the revised Rotterdam consensus (17, 18). Women with regular menstruation, normal ovarian function, and no clinical or biochemical profiles of hyperandrogenism served as controls. All participants were under 35 years old. Women with systemic diseases, endometriosis, or abnormal prolactin levels or thyroid function were excluded from this study.

### Clinical and biochemical measurements

A physical examination was performed, including weight, height, and body mass index (BMI, kg/m^2^). During the first 5 days of the menstrual cycle, fasting blood was collected for the measurement of fasting insulin (FINS), fasting plasma glucose (FPG), anti-Mullerian hormone (AMH), testosterone (T), follicle stimulating hormone (FSH), luteotropic hormone (LH), low-density lipoprotein (LDL), triglycerides (TG), total cholesterol (TC), and high-density lipoprotein (HDL). Homeostasis model assessment of insulin resistance (HOMA-IR) was calculated using a equation of FPG (mM) × FINS (mIU/L)/22.5 (18). Moreover, the antral follicle count (AFC) was determined by measuring the number of follicles 2–9 mm by transvaginal ultrasound.

### Follicular fluid collection and retrieval of ovarian granulosa cells

Ovarian stimulation and oocyte retrieval were performed using a previously described protocol (19). After adequate follicle development, human chorionic gonadotropin (hCG) was administered to trigger. Oocyte retrieval was performed 36 h after hCG injection, and ovarian granulosa cells were collected from the follicular fluid without blood contamination as described previously (20). Granulosa cells were purified with Ficoll-Paque (Solarbio, Beijing, China). Both granulosa cells and follicular fluid samples were stored at −80°C.

### Pregnancy complications

In this study, the patients' pregnancy complications included gestational diabetes, gestational hypertension, preterm delivery, and anemia. Definitions of these pregnancy complications were presented in previous articles (21–23).

### Quantification of miR-200c and miR-141 expression in human granulosa cells

TRIzol Reagent (Life Technologies, Shanghai, China) was used to extract total RNA from granulosa cells according to the manufacturer's instructions. Total RNA was reverse transcribed into cDNA using the MiRNA-X miRNA First-Strand Synthesis Kit (Takara-Clontech, China) following the manufacturer instructions. The primers used for RT-PCR were as follows: miRNA-200c-3p(5′–3′):TAATACTGCCGGGTAATGATGGA; miRNA-141-3p(5′–3′):TAACACTGTCTGGTAAAGATGG. MiRNA-U6 purchased from Takara was performed as the internal control. RT-PCR was performed in triplicate on a Light Cycler 480 system according to the manufacturer's instructions with the Quanti Nova SYBR Green PCR Kit (QIAGEN, Germany). Amplification specificity was assessed by melting curve analysis and the relative miRNA levels in each sample were calculated using the 2^−ΔΔCT^ method.

### Statistical analysis

Data were analyzed using SPSS 21.0 (SPSS, Chicago, IL, USA) and reported as mean ± standard deviation (SD). Kolmogorov–Smirnov test was used to determine whether data were normally distributed. Normally distributed variables were analyzed by Student *t*-test to determine statistical significance, while non-parametric data were assessed using the Mann-Whitney U-test. To avoid the effects of age and BMI on miR-141 and miR-200c expressions, logistic regression was used for adjusting. Categorical data were reported as percentage and frequency and analyzed by means of chi-square analysis, with the use of Fisher's exact test when expected frequencies were <5. A *p*-value <0.05 was considered statistically significant.

## Results

### Clinical characteristics of PCOS patients and controls

A total of 123 participants (62 PCOS patients and 61 controls) were included and their clinical characteristics were showed in Table 1. PCOS patients had significantly higher BMI, FINS, FPG, HOMA-IR, LH, AMH, T, LDL, TG, TC, and AFC (*p* < 0.05, for all), but HDL and FSH levels were lower than controls(*p* < 0.001 and *p* = 0.001, respectively). In terms of age, no difference was found between the two groups.

**Table 1:** Clinical and endocrine parameters in PCOS patients and controls.

**Basic parameters**	**PCOS (*n* = 62)**	**Control (*n* = 61)**	***p-*values**
Age (years)	28.27 ± 3.10	28.71 ± 2.46	0.362
BMI (kg/m^2^)	24.40 ± 3.34	21.77 ± 2.37	*p* < 0.001
FPG(mmol/L)	5.36 ± 0.47	5.19 ± 0.43	0.032
FINS(mIU/L)	14.50 ± 7.18	7.86 ± 1.94	*p* < 0.001
HOMA-IR	3.67 ± 2.07	1.80 ± 0.47	*p* < 0.001
LH (IU/L)	8.17 ± 3.84	4.90 ± 1.42	*p* < 0.001
FSH (U/L)	5.90 ± 1.13	6.52 ± 1.04	0.001
T(ng/dL)	36.75 ± 14.59	22.75 ± 7.48	*p* < 0.001
AMH (ng/ml)	9.40 ± 4.36	4.00 ± 1.83	*p* < 0.001
HDL (mmol/l)	1.28 ± 0.22	1.41 ± 0.20	*p* < 0.001
LDL (mmol/l)	3.07 ± 0.37	2.79 ± 0.59	*p* < 0.001
TG (mmol/l)	1.11 ± 0.45	0.79 ± 0.25	*p* < 0.001
TC (mmol/l)	4.51 ± 0.52	4.30 ± 0.66	0.041
AFC (mmol/l)	26.05 ± 7.98	12.91 ± 3.56	*p* < 0.001

*BMI, body mass index; FPG, fasting plasma glucose; FINS, fasting insulin; HOMA-IR, homeostasis model assessment of insulin resistance; FSH, follicle stimulating hormone; LH, luteotropic hormone; T, testosterone; AMH, anti-Mullerian hormone; HDL-C, high-density lipoprotein cholesterol; LDL-C, low-density lipoprotein cholesterol; TG, triglyceride; TC, total cholesterol*.

### Relative expression of miR-200c and miR-141

**Figure 1:**
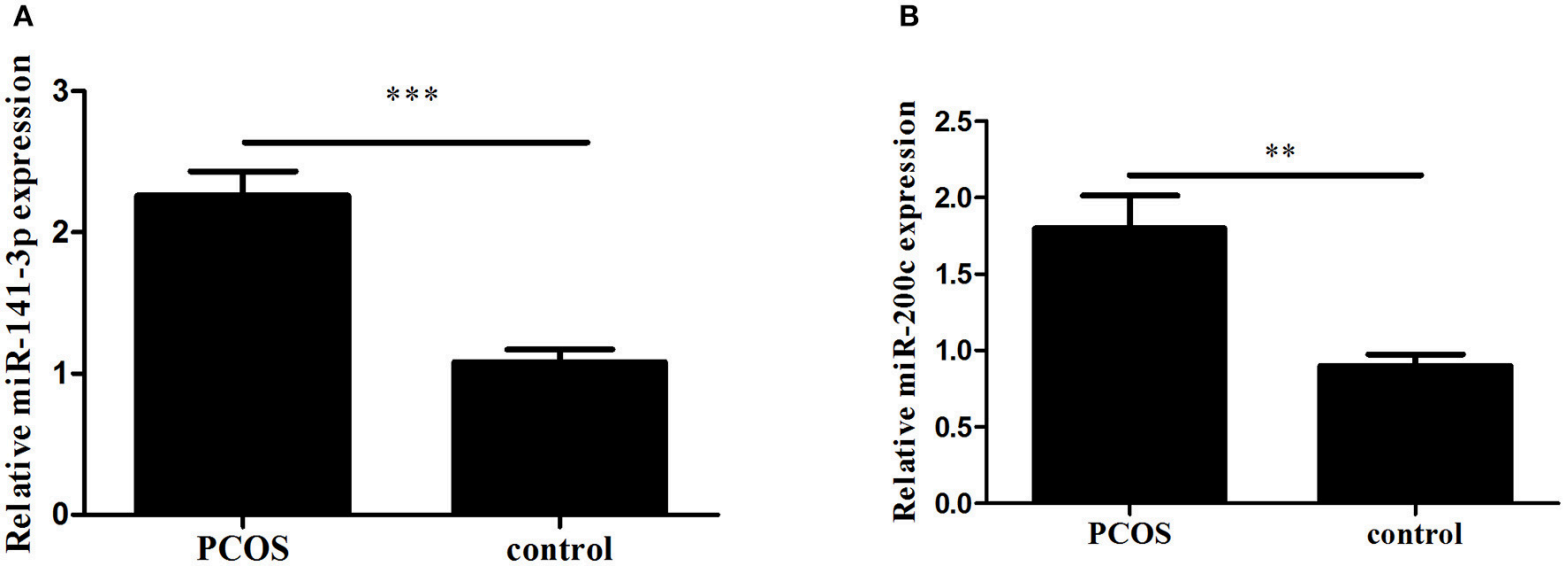
The expression of miR-141 and miR-200c in Polycystic Ovary Syndrome (PCOS) and control groups. Results are showed as the mean of (miRNAs of interest/U6) ± SD. **(A)** Expression of miR-141. **(B)** Expression of miR-200c. ***p < 0.001, **p = 0.002. PCOS women, *n* = 62; control women, *n* = 61. The groups were compared by Student *t*-test to determine statistical significance, A *p*-value < 0.05 was considered statistically significant.

Expression levels of miR-200c and miR-141 were examined by RT-PCR. After adjustment for age and BMI, the expressions of miR-200c and miR-141were significantly increased in PCOS group (*p* < 0.001, *p* = 0.002, respectively), and the results were showed in Figure 1. Compared with controls, PCOS patients had a significantly increased incidence of pregnancy complications in low-expression groups of miR-141 and miR-200c (*p* = 0.007 and *p* = 0.002, respectively), while there were no differences in the high-expression groups (Tables 2, 3).

**Table 2:** Comparison of pregnancy complications with different levels of miR-141.

**Groups**		**Pregnancy complications**	***p* (PCOS vs. controls)**
miR-141 low-expression group	PCOS (*n* = 25)	8	0.007
	Controls (*n* = 51)	3	
miR-141 high-expression group	PCOS (*n* = 37)	4	0.611
	Controls (*n* = 50)	2	

**Table 3:** Comparison of pregnancy complications with different levels of miR-200c.

**Groups**		**Pregnancy complications**	***p* (PCOS vs. controls)**
miR-200c low-expression group	PCOS (*n* = 28)	9	0.002
	Controls (*n* = 50)	2	
miR-200c high-expression group	PCOS (*n* = 34)	5	1
	Controls (*n* = 11)	1	

## Discussion

This was a pioneering study assessing the levels of miR-200c and miR-141 in granulosa cells derived from women with PCOS, particularly taking patients' age and BMI into consideration. We demonstrated that the levels of miR-200c and miR-141 were significantly increased in PCOS granulosa cells. In addition, compared with controls, PCOS patients had a significantly increased incidence of pregnancy complications in low-expression groups of miR-141 and miR-200c.

A previous report showed that transgenic mice expressing miR-141/200c developed a diabetic phenotype, while miR-200c-null mice had normal glucose tolerance, demonstrating that miR-141 and miR-200c played an important role in insulin resistance (24). As 50–70% women with PCOS are associated with insulin resistance, therefore, our findings that the levels of miR-200c and miR-141 were significantly increased in PCOS patients were in consistency with the above results. In rat granulosa cells, miR-141 may inhibit granulosa cells apoptosis via targeting *DAPK1*, further indicating that miR-141 might contribute to the pathogenesis of PCOS (14). It has been shown that both miR-141 and miR-200c participated in the regulation of multiple signaling pathways, including phosphatidylinositol-3-kinase (PI3K) and Wingless-type protein (Wnt) signaling pathways, and both of which were related to PCOS closely (25–29). The Wnt signaling pathway controlled a large variety of cellular processes, including cell proliferation, cell fate decision, pluripotency, and the establishment of cell polarity, while PI3K signaling pathway played an important role in regulating metabolic disorders, such as insulin resistance and diabetes (28, 30, 31). Abnormal follicle proliferation and metabolic dysregulation are typical characteristics of PCOS and it was concluded that miR-200c and miR-141 might influence the occurrence and development of PCOS through PI3K and Wnt signaling pathway (32).

In this study, we also found that in low-expression groups of miR-141 and miR-200c, PCOS patients had an increased risk of pregnancy complications. MiR-141 are known to play essential functions in regulating the process of pregnancy, ranging from embryo implantation and embryo development to fetal growth restriction (33–35). MiR-200c displays an altered expression in placentas from pregnancies complicated by preeclampsia and preterm labor, providing further evidence that miR-200c plays a crucial role in pregnancy (16). However, this was the first time that the altered expressions of miR-141 and miR-200c in granulosa cells were found to be associated with pregnancy complications. Our results, along with previous observations of significantly increased risk of pregnancy complications in PCOS, also suggested that typical characteristics of PCOS, such as hyperandrogenism, insulin resistance, obesity, and metabolic abnormalities, may lead to pregnancy complications (36, 37). However, the strategies for preventing occurrence and development of pregnancy complications in PCOS patients remain to be elucidated. Further research is needed to disclose pathophysiological mechanisms of pregnancy complications in PCOS.

In the meantime, this study had some limitations. First, the number of samples was relatively smaller than many previous expression studies and the results need to be verified by large sample size. Moreover, granulosa cells in this study were isolated from mature follicles, which might not represent the expression of miRNAs in immature follicles accurately.

Overall, our findings indicated that the levels of miR-200c and miR-141 were increased significantly in PCOS granulosa cells. PCOS patients had a significantly increased incidence of pregnancy complications in low-expression groups of miR-141 and miR-200c. This study provides new evidences about the underlying molecular mechanisms of PCOS.

## Author contributions

YS and HL designed the study. TH, YL, YJ contributed equally to the performance of the experiments. HW and XY performed data collection. TH drafted the manuscript. YL, YJ, HW, XY, GL, HL and YS proofread the manuscript.

### Conflict of interest statement

The authors declare that the research was conducted in the absence of any commercial or financial relationships that could be construed as a potential conflict of interest.
